# Simultaneous Spectrophotometric Determination of Lansoprazole and Domperidone in Capsule Dosage Form

**DOI:** 10.4103/0250-474X.40343

**Published:** 2008

**Authors:** A. P. Sherje, A. V. Kasture, K. N. Gujar, P. G. Yeole

**Affiliations:** Department of Pharmaceutical Chemistry, Sinhgad Institute of Pharmacy, 45-1/2/3, Narhe, Pune - 411 041, India; 1 Department of Institute of Pharmaceutical Education and Research, Borgaon (Meghe), Wardha - 442 001, India

**Keywords:** Lansoprazole, domperidone, spectrophotometry, simultaneous equation, formulation

## Abstract

Two simple, accurate and precise spectrophotometric methods have been developed for simultaneous determination of lansoprazole and domperidone in pharmaceutical dosage form. Method A involves formation of Q-absorbance equation at 256.0 nm (isoabsorptive point) and at 294.2 nm while method B is two wavelength method where 277.6 nm, 302.1 nm were selected as λ_1_ and λ_2_ for determination of lansoprazole and 231.3 nm, 292.0 nm were selected as λ_1_ and λ_2_ for determination of domperidone. Both the methods were validated statistically and recovery studies were carried out. The Beer's law limits for each drug individually and in mixture was within the concentration range of 5-50 μg/ml. Linearity of lansoprazole and domperidone were in the range of 24-36 μg/ml and 8-12 μg/ml, respectively. The proposed methods have been applied successfully to the analysis of the cited drugs either in pure form or in pharmaceutical formulations with good accuracy and precision. The method herein described can be employed for quality control and routine analysis of drugs in pharmaceutical formulations.

Lansoprazole (LAN), chemically 2-[[[3-methyl-4-(2,2,2-trifluoroethoxy)-2-pyridinyl] methyl] sulfinyl]-1H-benzimidazole is a proton pump inhibitor^1^–^3^. It is official in USP^2^ in which liquid chromatography is the method for assay. Other reports are available in the literature for determination of LAN from commercial dosage form and in biological samples including HPLC^4^–^5^, HPTLC^6^, LC-MS-MS^7^, spectrophotometry^8^–^9^. Domperidone (DOM), 5-chloro-1-[1-[2,3-dihydro-2-oxo-1H-benzimidazole-1-yl]propyl]-4-piperidyl]-2, 3-dihydro-1H-benzimidazol-2-one is a dopamine antagonist and indicated as antiemetic and antinauseant^10^. It is official in IP, BP and European Pharmacopoeia where non-aqueous titration is the official method^11^,^12^. Several methods are reported for determination of DOM individually or in combination with other drugs^13^–^15^. A fixed dose combination containing LAN and DOM is available commercially in the market as capsule dosage form and is indicated in acid related disorders. However there is no method reported for simultaneous estimation of these drugs in combined dosage form. Hence, an attempt has been made to develop simple, sensitive, accurate and precise analytical methods. The present communication describes two simple spectrophotometric methods for simultaneous estimation of these drugs from their combined formulation.

Reference standard of LAN was obtained from Dr. Reddy's Laboratories Ltd., Medak, India and DOM was obtained from Aurobindo Pharma Ltd., Hyderabad, India. All the reagents and chemicals were either of AR grade or spectroscopy grade. All the solutions were freshly prepared with double distilled water. Spectral absorbance measurements were made with Shimadzu UV-2401 double beam spectrophotometer with 1 cm matched quartz cell.

About 60 mg of LAN and 20 mg DOM were separately taken in a 100 ml volumetric flask, dissolved in a mixture of methanol and 0.1 M NaOH (70:30 v/v) and volume was made up to the mark. The standard stock solutions were further diluted separately to obtain a concentration of 30 μg/ml of LAN and 10 μg/ml of DOM. The resulting solutions were scanned in the range of 200-400 nm. The UV absorption overlain zero order spectrum for LAN and DOM is depicted in fig. 1. From the overlain spectra, the wavelengths 256.0 nm (isoabsorptive point) and 294.2 nm (λmax of LAN) were selected for formation of Q-absorbance equation. The standard stock solutions of these drugs were diluted to obtain a concentration range of 10-100 μg/ml and absorbances were measured at selected wavelengths. The concentrations of drugs against absorbance was plotted to obtain a calibration curve. Both the drugs obey Beer's law individually and in mixture within the concentration range of 5-50 μg/ml. The absorptivity values (A 1%, 1 cm) of each drug at selected wavelengths were determined. The concentration of each drug in laboratory mixture was determined by substituting the absorbance and absorptivity values in the following equations, Cx = (Qm-Qy/Qx-Qy) × A/Ax_1_ and Cy = (Qm-Qx/Qy-Qx) × A/Ay_1_^17^, where, Cx is the concentration of LAN, Cy is the concentration of DOM, Qm is the ratio of absorbance of sample at selected wavelengths, Qx is the ratio of absorptivity of LAN, Qy is the ratio of absorptivity of DOM, Ax_1_ is A (1%, 1 cm) of LAN at 256.0 nm, Ay_1_ is A (1%, 1 cm) of DOM at 256.0 nm.

**Fig. 1:**
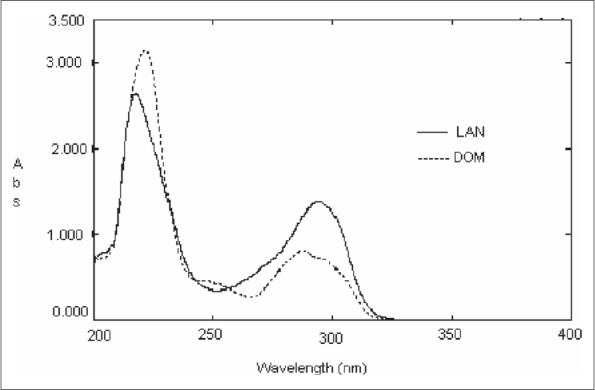
A typical overlain spectra of lansoprazole and domperidone. Zero order overlain spectra of lansoprazole and domperidone where (–) indicates UV absorbance spectrum of lansoprazole (LAN) and (----) denotes UV absorbance spectrum of domperidone (DOM).

The prior criteria for two wavelength method (method B) are existence of two such wavelengths where interfering component shows same absorbance whereas component of interest shows significant difference in absorbance. Based on this criterion, two wavelengths 277.6 nm and 302.1 nm were selected as λ_1_ and λ_2_ for estimation of LAN at which DOM shows same absorbance but LAN shows significant difference in absorbance. Similarly, wavelengths 231.3 nm and 292.0 nm were selected as λ_1_ and λ_2 for_ estimation of DOM. For calibration curve, the standard stock solutions of these drugs were diluted in the concentration range of 10-100 μg/ml and absorbances were recorded at selected wavelengths. Both the drugs obey Beer's law individually and in mixture within the concentration range of 5-50 μg/ml. From standard stock solutions five laboratory mixtures (samples) and one as standard were prepared containing 30 μg/ml of LAN and 10 μg/ml of DOM. The absorbance of the resulting solutions were measured at the selected wavelengths and concentration of each drug was determined using the following equation, Cu = Au/As x Cs/Cu, where, Cu is the concentration of unknown, Cs is the concentration of standard, Au is the absorbance of unknown, As is the absorbance of standard and d is the dilution factor.

For analysis of capsule formulation, twenty capsules (Leedom manufactured by Bestochem Formulation (I) Ltd. and Lans-DX manufactured by Zydus Recon Healthcare Ltd. India) were weighed, contents removed and finely powdered. For method A, quantity of powder equivalent to 30 mg of LAN and 10 mg of DOM was weighed accurately and to it 20 mg of pure DOM was added (standard addition method). The mixture was dissolved in solvent and filtered with 0.45 μ membrane filter paper. An aliquot of filtrate was pipetted and diluted to obtain concentrations 30 μg/ml of LAN and 10 μg/ml of DOM. The absorbance of resulting solutions was measured at selected wavelengths. For method B, a calibration curve of seven mixed standards was prepared by plotting the concentrations of drugs against absorbance difference at selected wavelengths. A quantity of powder equivalent to 30 mg of LAN and 10 mg of DOM was weighed accurately, dissolved in solvent and filtered. The filtrate was diluted further to obtain a concentration of 30 μg/ml of LAN and 10 μg/ml of DOM. The absorbances of the resulting solutions were recorded at the selected wavelengths and concentration of each drug was obtained by extrapolating the absorbance value on standard calibration curve of mixed standards.

The recovery studies were carried out at different concentrations by spiking a known concentration of standard drug to the pre-analyzed sample and contents were reanalyzed by proposed methods. The results of marketed formulation analysis and recovery studies are depicted in Table 1. The methods were validated statistically as per ICH/USP^16^ guidelines for parameters like accuracy, precision, ruggedness, specificity, linearity and range (Table 2). Accuracy was ascertained on the basis of recovery studies. Precision was studied by analyzing five replicates of sample solutions and concentrations were calculated. Ruggedness was established by carrying out experiment at different conditions like intra day, inter day and by different analyst. Specificity of methods was ascertained by analyzing the solutions under different stress conditions like basic (0.1 N NaOH, 1.0 ml, 40°), oxidation (3% v/v H_2_ O_2_, 1.0 ml, 40°), heat (60°) for 24 h. Linearity and range were determined by analyzing 80-120% of test concentrations of each drug.

**TABLE 1:** RESULTS OF CAPSULE FORMULATION ANALYSIS AND RECOVERY STUDIES

Method	Drug	Label claim (mg/capsule)	Amount found (mg)	% Drug found (Mean ± SD), *n* = 3	% Recovery (Mean ± SD), *n* = 3
A	LAN	30	30.10	100.34 ± 0.5556	99.63 ± 0.6341
	DOM	10	10.00	100.02 ± 0.5699	99.85 ± 0.5489
B	LAN	30	29.86	99.55 ± 0.4903	99.61 ± 0.5435
	DOM	10	09.98	99.83 ± 0.7178	99.66 ± 0.4225

Method A is Q-absorbance method while method B is two wavelength method. Results are mean of three determinations (*n* = 3), SD is standard deviation, LAN denotes lansoprazole and DOM denotes domperidone

**TABLE 2:** OPTICAL CHARACTERISTICS AND VALIDATION OF THE PROPOSED METHODS

Parameters	Method A		Method B	
		
	LAN	DOM	LAN	DOM
Linearity range (μg/ml)	24-36	8-12	24-36	8-12
Beer's law limit (μg/ml)	5-50	5-50	5-50	5-50
Intercept	0.043^a^, 0.0117^b^	0.0234^a^, 0.0117^b^	0.0018^c^	0.0128^d^
Slope	0.0359^a^, 0.005^b^	0.0136^a^, 0.004^b^	0.0138^c^	0.022^d^
Correlation coefficient (r)	0.9994^a^, 0.9985^b^	0.9993^a^, 0.9972^b^	0.9995^c^	0.9997^d^
Accuracy (% Recovery)	99.63	99.85	99.61	99.66
Precision (RSD, *n* = 5)	0.5556	0.5699	0.4903	0.7178
Ruggedness (% Label claim, *n* = 3)				
Intra-day	99.73	99.59	100.08	99.85
Inter-day	99.98	100.34	99.85	100.30
Different analyst	99.79	99.70	100.08	99.78
Specificity	Specific	Specific	Specific	Specific

In the above table ‘a’ indicates results at 294.2 nm; ‘b’ at 256.0 nm; ‘c’ at 277.6 nm and 302.1 whereas, ‘d’ denotes results at 231.3 nm and 292.0 nm. Method A is Q-absorbance method while method B is two wavelength method. LAN is lansoprazole and DOM is domperidone, RSD is relative standard deviation

In the proposed method for analysis of LAN and DOM in commercial formulation a mixture of methanol and 0.1 M NaOH is used as the solvent. The overlain spectrum of LAN and DOM does not give any suitable isoabsorptive point in a concentration proportion of 3:1 respectively whereas the overlain spectra of both drugs in 1:1 ratio (30 μg/ml of each drug) shows a reproducible isoabsorptive point at 256.0 nm. Hence standard addition technique was employed in order to bring a concentration ratio of 1:1 (30 μg/ml). Thus estimation of drugs by Q- absorbance equation method (method A) was carried out at 256.0 nm (isoabsorptive point) and 294.2 nm (λmax of LAN). Method B involves four wavelengths for estimation of two drugs. The wavelengths 277.6 nm and 302.1 nm were selected for estimation of LAN where DOM shows same absorbance but LAN shows significant difference in absorbance whereas 231.3 nm and 292.0 nm satisfies the criteria for estimation of DOM. The proposed methods were successfully used to estimate LAN and DOM in marketed capsule formulation. The assay values were in good agreement with the corresponding labeled claim. The recovery studies show accuracy of the method. On observing the validation parameters both the methods were found to be accurate, precise and specific. Hence the methods can be employed for quality control and routine analysis of lansoprazole and domperidone in pharmaceutical formulations.

## References

[1] Sweetman SC (2005). Martindale-The Complete Drug Reference.

[2] USP 28- NF 23 (2005).

[3] Keith GT, Gennaro AR (2000). Gastrointestinal and liver drugs. Remington: The Science and Practice of Pharmacy.

[4] Unoa T, Yasui FN, Takahata T, Sugawara K, Tateishi T (2005). determination of lansoparazole and two of its metabolites by liquid-liquid extraction and automated column-witching high-performance liquid chromatography: Application to measuring CYP2C19 activity. J Chromatogr B Anal Technol Biomed Life Sci.

[5] Avgerinos A, Karidas T, Potsides C, Axarlis S (1998). Determination of lansoprazole in biological fluids and pharmaceutical dosage by HPLC. Eur J Drug Metab Pharmacokinet.

[6] Pandya KK, Mody VD, Satia MC, Mody A, Mody RK, Chakravarthy BK (1997). High performance thin layer chromatographic method for detection and determination of lansoprazole in human plasma and its use in pharmacokinetic studies. J Chromotoqr B Biomed Sci Appl.

[7] Celso H, Oliveiraa R, Barrientos-Astagarragab E, Eduardo A, Gustavo D, Mendesb D (2003). Lansoprazole quantitation in human plasma by liquid chromatography-electrospray tandem mass spectrometry. J Chromatogr B.

[8] Puratchikodi A, Krishnamoorthy G, Joykat B, Valarmathy R (1996). Spectrometric method for the determination of lansoprazole. Eastern Pharmacist.

[9] Wahbi AA, Omatna AR, Azzig A, Hada M, Matwa SM (2002). Spectrometric determination of omeprazole, lansoprazole and pantoprazole in pharmaceutical formulations. J Pharm Biomed Anal.

[10] Altman DF, Katzung BG (2001). Basic and clinical Pharmacology.

[11] (1996). British Pharmacopoeia.

[12] (1997). European Pharmacopoeia.

[13] Smit MJ, Sutherland FC, Hundt HK, Swart KJ, Hundt AF (2002). Rapid and sensitive liquid chromatography-tandem mass spectrometry method for the quantitation of domperidone in human plasma. J Chromatogr A.

[14] Vinodhlni C, Vaidhyalingam V, Ajithadas A, Niraimathi, Shantha A (2002). Simultaneous estimation of cinarizine and domperidone in solid oral dosage form using Spectrophotometric method. Indian Drugs.

[15] Manoj K, Anbazhagan S (2004). Reverse phase high performance liquid chromatographic method for simultaneous estimation of domperidone and pantoprazole from tablet formulation. Indian Drugs.

[16] Rockville MD, USP 28- NF 23 (2005). United States Pharmacopeial Convention, Inc.

[17] Davidson AG, Beckett AH, Stenlake JB (2004). Ultraviolet-visible absorption spectrophotometry. Practical Pharmaceutical Chemistry.

